# Association Between Point-of-Care Viral Testing for Influenza and Adenovirus and Antibiotic Management in a Pediatric Emergency Department in Italy

**DOI:** 10.3390/children13010151

**Published:** 2026-01-21

**Authors:** Tommaso Bellini, Andrea Lacovara, Daniele Franzone, Marcello Mariani, Giorgia Iovinella, Martina Virgilio, Julia Lasagna, Simona Matarese, Carlotta Pepino, Francesca Canzoneri, Milena Guazzi, Emanuela Piccotti, Andrea Moscatelli

**Affiliations:** 1 Paediatric Emergency Room and Emergency Medicine Unit, Department of Emergency Medicine, Anesthesia and Critical Care, Istituto di Ricerca e Cura a Carattere Scientifico Istituto Giannina Gaslini, 16147 Genoa, Italy; danielefranzone@gaslini.org (D.F.); simonamatarese@gaslini.org (S.M.); carlottapepino@gaslini.org (C.P.); francescacanzoneri@gaslini.org (F.C.); milenaguazzi@gaslini.org (M.G.); emanuelapiccotti@gaslini.org (E.P.); 2 Department of Neuroscience, Rehabilitation, Ophthalmology, Genetics, Maternal and Child Health (DINOGMI), University of Genoa, 16148 Genoa, Italy; 5727889@studenti.unige.it (A.L.); 3672473@studenti.unige.it (G.I.); 6554088@studenti.unige.it (M.V.); 4245232@studenti.unige.it (J.L.); 3 Infectious Diseases Unit, Department of Pediatrics, Istituto di Ricerca e Cura a Carattere Scientifico Istituto Giannina Gaslini, 16147 Genoa, Italy; 4 Pediatric and Neonatal Intensive Care Unit, Department of Emergency Medicine, Anesthesia and Critical Care, Istituto di Ricerca e Cura a Carattere Scientifico Istituto Giannina Gaslini, 16147 Genoa, Italy; andreamoscatelli@gaslini.org

**Keywords:** antibiotic stewardship, adenovirus, clinical decision-making, emergency care, influenza, pediatrics, point-of-care testing, respiratory tract infections

## Abstract

**Highlights:**

**What are the main findings?**

- Rapid Diagnostic Test (RDT) positivity for influenza or adenovirus was associated with lower antibiotic prescription and higher discontinuation of pre-existing antibiotic therapy in children with febrile respiratory illness.
- Antibiotic prescription or discontinuation decisions made after RDT results were not associated with higher 72-h return rates.

**What are the implications of the main findings?**

- Bedside viral RDTs may support diagnostic assessment and antibiotic decision-making in clinically stable children.
- Wider adoption of RDTs may contribute to optimizing antibiotic use in acute pediatric settings but their effectiveness on AMR requires further dedicated studies.

**Abstract:**

**Background:** Respiratory tract infections (RTIs) represent one of the most prevalent reasons for visits to Pediatric Emergency Departments (PEDs). Because viral and bacterial presentations frequently overlap, a substantial proportion of antibiotic prescriptions in pediatric acute care are potentially unnecessary, contributing to antimicrobial resistance. Rapid Diagnostic Tests (RDTs) for respiratory viruses have been suggested as tools to enhance diagnostic precision and support antimicrobial stewardship. However, evidence regarding their real-world impact in pediatric emergency settings is limited. **Objectives:** This study aimed to assess the association between point-of-care RDT results and antibiotic management in a tertiary PED, focusing on both the discontinuation of antibiotics in children already receiving treatment and the avoidance of new antibiotic prescriptions in untreated children. The secondary objective was to evaluate the short-term safety through 72-h return visits. **Methods**: A retrospective cohort study was conducted at a tertiary PED during two epidemic seasons (December–February 2023–2024 and 2024–2025). Children aged <18 years who underwent RDTs for febrile respiratory illnesses were included. Patients were stratified based on whether they were already receiving antibiotic therapy at presentation. The primary outcomes were antibiotic discontinuation among treated patients and initiation among untreated patients. Unplanned return visits to the PED within 72-h post-discharge were used as a pragmatic short-term safety outcome to capture early clinical deterioration. RDTs (SD Biosensor Standard F Antigen) were performed at the bedside with a turnaround time of 10–15 min. **Results**: A total of 1238 children were included, of whom 330 (26.6%) tested positive for influenza and/or adenovirus. Among the 234 children already receiving antibiotics, discontinuation was significantly more frequent in the RDT-positive group (*p* < 0.001; OR 0.044). Among the 1004 untreated children, antibiotic prescription was significantly lower in the positive group than in the negative group (*p* < 0.001; OR 0.097). Return visits within 72-h did not differ between the groups in either cohort. No invalid tests occurred. **Conclusions**: Influenza/adenovirus RDT positivity was associated with lower antibiotic initiation among untreated children and higher discontinuation among those already receiving antibiotics, with no differences in 72-h return visits. These findings suggest a potential role for bedside viral testing as a decision-support tool for antibiotic management in the PED.

## 1. Introduction

Respiratory tract infections (RTIs) are among the most prevalent illnesses in childhood and constitute a primary reason for healthcare utilization and visits to Pediatric Emergency Departments (PEDs) [1,2,3,4]. School-aged children typically experience three to ten fever-associated respiratory illnesses annually, indicating the substantial burden of viral circulation in early life [5,6]. Seasonal influenza, parainfluenza viruses, respiratory syncytial virus, and adenovirus are responsible for most RTIs; however, their clinical manifestations often overlap with those of bacterial infections, complicating etiological differentiation [3,6]. This diagnostic uncertainty, along with time constraints and limited longitudinal follow-up in PEDs, contributes to the inappropriate use of antibiotics, which remains a significant driver of antimicrobial resistance (AMR) globally [2,3,5,6,7,8,9,10,11].

It is estimated that up to 50% of antibiotic prescriptions in pediatric settings are clinically unnecessary, with Italy historically ranking among the European nations with the highest rates of antibiotic consumption and bacterial resistance [4,5,12,13,14,15]. Given the increase in AMR and the prevalence of viral etiologies in young children, enhancing diagnostic accuracy during the acute phase of respiratory illnesses is crucial for optimizing antibiotic use and implementing effective antimicrobial stewardship strategies [12,13,16,17]. In this context, hospital-based antimicrobial stewardship evidence demonstrates that structured, data-driven approaches can safely reduce unnecessary antimicrobial use while maintaining clinical outcomes [18]. Moreover, because emergency care is responsible for a relevant share of acute antibiotic prescriptions, improving diagnostics at this point of care is necessary to mitigate antimicrobial resistance. Traditionally, etiological diagnosis has depended on molecular assays such as multiplex real-time polymerase chain reaction (RT-PCR), which offer high sensitivity and specificity but are constrained by laboratory processing requirements and turnaround times (TAT) of several hours [2,7,19,20]. To address these limitations, Point-of-Care Rapid Diagnostic Tests (RDTs) have been increasingly utilized in emergency and ambulatory care settings [11,12,19,21]. These tests provide results within minutes, can be administered at the bedside by minimally trained personnel, and have been associated with improved diagnostic certainty, antiviral prescribing, and lower antibiotic use in specific contexts [5,12,21].

Evidence on the real-world impact of bedside influenza/adenovirus RDTs on both antibiotic initiation and discontinuation in PED settings is limited. Thus, the present study aimed to evaluate the clinical implications of RDTs for influenza A/B and adenovirus in this context, and their role in advancing pediatric antimicrobial stewardship while ensuring clinical safety. Specifically, it examines whether early viral identification correlates with a reduction in the initiation of antibiotic therapy or facilitates the safe discontinuation of antibiotics prescribed prior to PED evaluation. Furthermore, this study evaluated the safety of RDT-guided management by analyzing return visits to the PED within 72-h post-discharge.

## 2. Materials and Methods

A retrospective observational cohort study was conducted at the PED of the Istituto di Ricerca e Cura a Carattere Scientifico (IRCCS) Giannina Gaslini, focusing on pediatric patients who presented to the PED during the last two epidemic seasons (2023–2024, 2024–2025). These seasons were defined as the period from the first day of December to the last day of February, according to the local peak circulation of influenza and the main period of PED admission due to respiratory illnesses. The IRCCS Giannina Gaslini is a tertiary care children’s hospital located in the Liguria region of northwestern Italy, with an annual average of 38,000 PED visits by patients aged 0–17 years. The inclusion criteria comprised all consecutive patients who underwent RDT for adenovirus and/or influenza due to febrile illness with respiratory symptoms. According to local practice, RDTs were available for febrile respiratory presentations; however, test ordering ultimately remained at the treating physician’s clinical judgment. Patients presenting with hemodynamic instability, signs of sepsis, respiratory failure, or other critical conditions requiring immediate life-saving interventions were not eligible for rapid diagnostic testing and were therefore excluded from the study population. Therefore, the study population consisted exclusively of clinically stable children with fever and respiratory symptoms. The decision to perform RDTs for influenza only, adenovirus only, or both was ultimately at the physician’s discretion, influenced by the epidemiological context or specific associated clinical features of the disease. Patients were classified as ‘positive’ if at least one RDT yielded a positive result, even when multiple tests were conducted. Patients with negative results for all tests were included in the ‘negative’ group. Influenza and adenovirus were selected among all viruses because of their availability as bedside RDTs in our PED and the possibility of interpreting the data obtained directly from the device used to read the RDTs. Moreover, they were grouped to reflect shared clinical management and antibiotic stewardship decisions.

**Figure 1:**
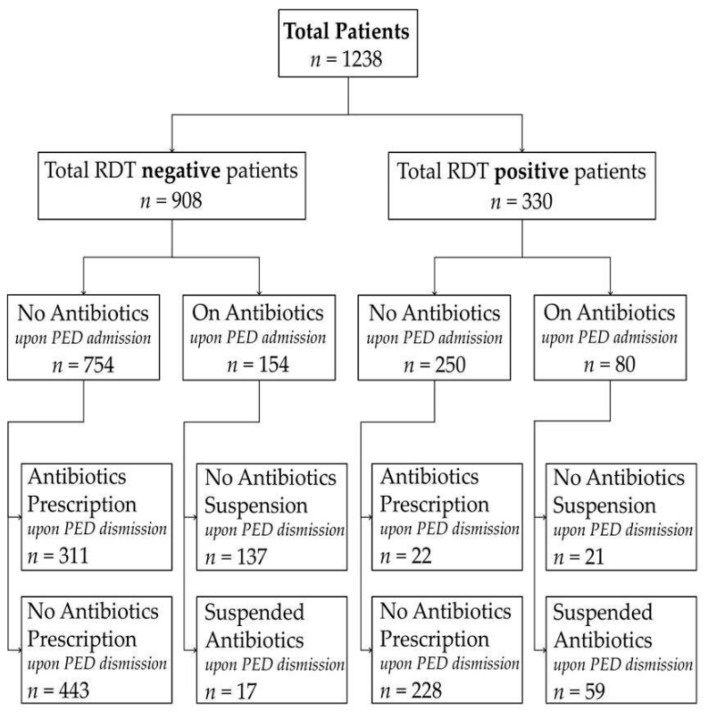
Flowchart of patient selection and antibiotic management according to rapid diagnostic test (RDT) results. The figure illustrates the study cohort stratified by RDT result (positive vs. negative) and antibiotic status at pediatric emergency department (PED) admission (on antibiotics vs. not on antibiotics). For each subgroup, antibiotic management at PED discharge (initiation, continuation, or suspension) is reported. All antibiotic decisions refer to discharge management and were made after availability of bedside RDT results.

Patients were stratified into two groups: those who had already received antibiotic therapy at the time of presentation to the PED and those who had not received antibiotics prior to the PED evaluation. All retrospective data were collected from anonymized electronic medical records. Demographic characteristics, including age, sex, return visits to the PED within 72 h post-discharge, and antibiotic-related outcomes, were analyzed for each group. For patients already receiving antibiotic therapy, we assessed the rate of antibiotic suspension at PED discharge and the frequency of readmission among those whose antibiotics were suspended. A 72-h cutoff for unplanned return visits was predefined based on previous evidence [22,23]. Among patients not on antibiotics, we evaluated the rate of new antibiotic prescriptions and the frequency of readmission among those discharged without antibiotic therapy. When multiple RDTs were performed, the tests were conducted sequentially during the same PED visit, with unchanged clinical conditions owing to the short TAT. Antibiotic prescribing or discontinuation outcomes refer to discharge decisions made after bedside RDT results. Figure 1 shows how the enrolled patients were grouped. In addition, separate exploratory analyses were performed for influenza and adenovirus to evaluate whether the observed associations between rapid diagnostic test (RDT) positivity and antibiotic management were consistent across individual viral pathogens. These analyses were conducted by stratifying patients according to virus type and antibiotic status at pediatric emergency department admission.

All tests were conducted using the Standard F Antigen Point-of-Care produced by Relab, SD BiosensorTM, Genoa, Italy. The sensitivity and specificity for influenza were 97.0% and 97.6%, respectively, while those for adenovirus were 83.3% and 95.5%, respectively [11]. Tests have a TAT of 10 to 15 min and are performed on demand at the bedside, eliminating the need for transport to a laboratory. The point-of-care test requires no specific expertise and can be performed by healthcare personnel following a brief demonstration. All point-of-care tests were performed at the bedside by collecting a nasal swab sample by trained medical or nursing personnel, depending on staff availability during patient assessment.

### Statistical Analysis

Categorical variables are reported as absolute frequencies and percentages, and continuous variables are presented as medians and interquartile ranges. To evaluate differences between groups, the Kruskal–Wallis test or Mann–Whitney U-test was employed for continuous variables, and the chi-square or Fisher’s exact test was utilized for categorical variables. For categorical outcomes, odds ratios (ORs) with 95% confidence intervals (CIs) were calculated according to the Wald method. Statistical significance was set at *p* < 0.05, with all values determined using two-tailed tests. All statistical analyses were performed using GraphPad Prism version 9.1.0 for Windows (GraphPad Software, San Diego, CA, USA, www.graphpad.com, accessed on 15 December 2025) or IBM SPSS Statistics for Windows Version 21.0 (IBM Corp., Armonk, NY, USA) according to the test-specific needs. Key results were cross-checked for consistency.

## 3. Results

During the study period, 1238 patients satisfied the inclusion criteria, accounting for 6.5% of the 19,328 patients evaluated at our PED. Of these, 330 patients (26.6%) tested positive for adenovirus or influenza, while 908 patients (73.4%) tested negative. Of the 330 positive patients, 263 (79.7%) were tested positive for influenza and 67 (20.3%) for adenovirus, respectively. No invalid or failed RDTs were recorded according to the internal quality control criteria of the device. A total of 332 patients were tested for both viruses. Moreover, 234 patients were already receiving antibiotic therapy upon PED admission, whereas 1004 patients were not.

Among the 234 patients who had received antibiotics prior to PED admission, 80 (34.2%) presented a positive RDT, while 154 (65.8%) tested negative. There were no significant differences in sex distribution or median age between these groups. The suspension of antibiotics was significantly more prevalent among those with positive test results (*p* < 0.001; OR: 0.044, CI95%: 0.022–0.090). Among patients whose antibiotics were suspended, the readmission rate did not differ significantly.

Among the 1004 children who were not undergoing antibiotic therapy at the time of presentation to the PED, 250 (24.9%) tested positive, and 754 (75.1%) tested negative. The groups were comparable in terms of sex and age. Notably, the prescription of antibiotics was significantly less frequent among those who tested positive (*p* < 0.001; OR: 0.097, CI95%: 0.061–0.153). Among the children discharged without antibiotic therapy, the readmission rate did not differ significantly between those who tested positive and those who tested negative. Overall, RDT positivity was associated with a statistically lower rate of antibiotic prescription and with a statistically higher rate of antibiotic discontinuation at discharge, with a comparable 72-h readmission rate. Table 1 and Table 2 provide a summary of the main findings. When analyses were stratified by virus type, similar associations were observed for both influenza and adenovirus. In virus-specific analyses, RDT positivity remained associated with lower antibiotic initiation among untreated children and higher antibiotic discontinuation among those already receiving antibiotics, with no significant differences in 72-h return visit rates. These virus-specific analyses were planned as secondary exploratory analyses and are reported in the Supplementary Tables S1–S4.

## 4. Discussion

In this retrospective cohort study, RDT positivity for influenza A/B and adenovirus was associated with lower antibiotic use in a high-volume tertiary PED. By categorizing patients based on their ongoing antibiotic therapy at presentation, the study examined both antibiotic discontinuation among treated patients and antibiotic initiation among untreated patients. Across both patient groups, RDT positivity was associated with higher discontinuation among children already receiving antibiotics and lower initiation among untreated children. Similarly, among children already receiving antibiotics, a positive result strongly favored discontinuation. These patterns may reflect clinicians’ higher likelihood of attributing symptoms to viral etiology when RDTs were positive. In children not previously exposed to antibiotics, RDT positivity showed an association with lower antibiotic initiation among untreated patients, aligning with the growing body of pediatric evidence demonstrating that targeted viral identification has been associated with unnecessary antibiotic exposure [3,5,15,16]. Importantly, 72-h return visit rates were similar between groups, suggesting no signal of increased short-term revisits in this cohort. However, it should be noted that the RDT-negative group may represent a biologically heterogeneous population. This group likely includes children with other viral infections, and antibiotic decisions in RDT-negative cases may also have been influenced by additional clinical or laboratory information beyond viral testing. The observed associations were consistent when influenza and adenovirus were analyzed separately, suggesting that the main findings were not solely driven by pathogen aggregation but reflected similar patterns across different viral etiologies. Although these virus-specific analyses were limited by sample size, the consistency of the observed associations across influenza and adenovirus supports the internal coherence of the results and reduces concerns that pathogen grouping alone drove the main findings. Moreover, these findings align with pediatric data, indicating that rapid influenza tests have been associated with lower antibiotic prescriptions and reduced use of additional diagnostics in several studies in acute care settings [5,7,12,21,24]. The clinical utility of RDTs is well documented, particularly given the substantial global disease burden, which is notably high in children under five years of age [6]. Furthermore, the extensive genetic variability of influenza viruses, resulting from frequent mutations and epistatic interactions, complicates the clinical differentiation between viral and bacterial infections, thereby underscoring the importance of timely etiological confirmation [8]. However, influenza testing should not be the only focus of diagnostics. Although it is the most frequently utilized rapid viral test in pediatric emergency contexts, our findings suggest that adenovirus RDTs positivity showed a similar association with antibiotic management [5]. Current evidence supports the substantial stewardship benefits of influenza RDTs, while the clinical utility of testing for other viruses remains less clearly defined and requires further pediatric-focused research [4,7,24]. Our results report that adenovirus RDT positivity showed a similar association with antibiotic management. This may suggest a potential contribution to stewardship decisions, although causality cannot be inferred.

Notably, the impact of RDTs on the pediatric population appears to differ from that observed in adults. Numerous studies involving adults have reported enhanced viral detection and expedited TATs without significant reductions in overall antibiotic prescribing [25,26,27,28,29]. This discrepancy likely reflects variations in baseline prescribing patterns, comorbidity burdens, and clinical thresholds for initiating antibiotic treatment. Children generally exhibit a higher prevalence of viral diseases, fewer confounding factors, and more distinct syndromic patterns, rendering viral test results more actionable and reliable. Our findings underscore the necessity of refraining from extrapolating adult data to the pediatric population [29]. The significance of these findings is heightened in the context of the global AMR crisis. The burden of AMR remains substantial and is anticipated to increase, with notable geographic and age-related disparities [9,10]. Inappropriate antibiotic use, such as over-prescription, prolonged courses, and unnecessary broad-spectrum therapy, continues to be the primary driver of resistance [15,16,17]. Italy exhibits high rates of outpatient antibiotic prescriptions and considerable regional variability [14]. In this context, the use of RDTs may support antibiotic decision-making and could be evaluated within broader stewardship initiatives.

Antibiotic prescriptions in the emergency department are inherently multifactorial and based on overall clinical judgment; however, the integration of RDTs may have significant implications beyond this setting. Evidence from pediatric primary care suggests that rapid influenza testing has been associated with improved diagnostic certainty and lower antibiotic prescribing in some primary care studies during periods of peak viral circulation [3,30]. Although this study focused on PED, similar benefits may extend to community-based settings, particularly among younger children, where differentiating between viral and bacterial infections poses a considerable challenge. Thus, RDTs should not be used in isolation but should be integrated into the overall clinical assessment. In a population of clinically stable children with comparable respiratory presentations, viral RDT positivity may function as a decision-support tool and may be associated with a lower likelihood of prescribing antibiotics.

### Limitations

This study had several limitations that should be considered. First, its retrospective, single-center design constrains the generalizability of the findings to other epidemiological or organizational contexts. Moreover, comorbidities and vaccination status were not included as confounding factors because they were not available. Second, because RDTs were performed at the clinician’s discretion, patients undergoing testing likely represented a population in whom viral etiology was already suspected. Therefore, the observed association between test positivity and antibiotic management may also reflect pre-test clinical judgment rather than the independent effect of the test itself. Moreover, the biological heterogeneity of the RDT-negative group and the absence of systematic bacterial testing may have limited the interpretability of the antibiotic decisions in this subgroup. Third, RDTs were administered by trained medical or nursing personnel, contingent on availability, which may have introduced minor operational variations. However, no invalid tests were recorded, indicating a high procedural reliability. Fourth, excluding critically ill presentations may have biased the cohort toward lower-risk cases and limited generalizability to unstable children, in whom antibiotic decisions follow different thresholds. Moreover, no multivariable analysis was performed to adjust for potential confounders, such as age, illness severity, or prior testing, which limits causal inference. Finally, the 72-h readmission rates were low across all groups, which may have limited the statistical power to detect rare adverse outcomes associated with antibiotic discontinuation or withholding. Antimicrobial resistance outcomes and long-term safety beyond 72-h were not assessed.

## 5. Conclusions

In this single-center retrospective cohort, point-of-care influenza and adenovirus RDT positivity was associated with lower antibiotic prescription among untreated children and higher discontinuation among those already receiving antibiotics, without differences in short-term return visits. Given the observational design and non-standardized testing strategy, these findings should be interpreted as associative and may partly reflect pre-test clinical decision-making. Nevertheless, they suggest that bedside viral testing may support antibiotic management decisions in clinically stable pediatric patients in emergency settings. Future prospective multicenter studies with standardized testing algorithms and multivariable adjustments are needed to confirm these findings and the integration of RDTs into clinical decision support tools.

## Figures and Tables

**Table 1:** Main results comparing positive and negative tests in patients already on antibiotic therapy upon PED admission.

	Total Tested Patients on Antibiotics n = 234	RDTs Positive Patients n = 80	RDTs Negative Patients n = 154	*p*
Sex, m (%)	131 (56.0)	42 (52.5)	89 (57.8)	0.43
Age (years), median (IQR)	3.9 (2.1–6.9)	3.1 (2.4–6.9)	2.1 (1.7–9.1)	0.97
Suspended antibiotic therapy, yes (%)	76 (32.5)	59 (73.5)	17 (11.0)	<0.001
Readmission, yes (%) *	2/76 (2.5)	1/59 (1.5)	1/17 (5.5)	0.34

* of the total number of patients whose antibiotic therapy was suspended.

**Table 2:** Antibiotic prescription at discharge and 72-h readmission comparing positive and negative tests in patients without antibiotic therapy upon PED admission.

	Total Tested Patients Without Antibiotics n = 1004	RDTs Positive Patients n = 250	RDTs Negative Patients n = 754	*p*
**Sex,** ** m (%)**	590 (58.7)	149 (59.6)	441 (58.5)	0.75
**Age (years),** **median (IQR)**	3.1 (1.7–7.0)	2.9 (1.8–6.7)	2.7 (1.2–7.1)	0.20
**Patients dismissed with antibiotic prescription, yes (%)**	333 (33.0)	22 (9.0)	311 (41.0)	<0.001
**Readmission, ** yes (%) *	40/671 (6.0)	13/228 (5.5)	27/443 (6.0)	0.83

* of the total number of patients discharged without receiving antibiotic therapy.

## Data Availability

The datasets used and analyzed in this paper are available from the corresponding author on reasonable request. The data are not publicly available due to ethical and privacy-related restrictions.

## Supplementary Materials

The following supporting information can be downloaded at: https://www.mdpi.com/article/10.3390/children13010151/s1, Table S1. Antibiotic prescription at discharge and 72-h readmission according to adenovirus rapid diagnostic test results in patients not receiving antibiotic therapy at PED admission. Comparison of demographic characteristics, antibiotic prescription at discharge, and unplanned pediatric emergency department (PED) return visits within 72 h between adenovirus rapid diagnostic test (RDT)–positive and RDT-negative patients who were not receiving antibiotic therapy at presentation. Readmission rates are calculated among patients discharged without antibiotic therapy. Table S2. Antibiotic discontinuation and 72-h readmission according to adenovirus rapid diagnostic test results in patients receiving antibiotic therapy at PED admission. Comparison of demographic characteristics, antibiotic discontinuation at discharge, and unplanned pediatric emergency department (PED) return visits within 72 h between adenovirus rapid diagnostic test (RDT)–positive and RDT-negative patients who were receiving antibiotic therapy at presentation. Readmission rates are calculated among patients whose antibiotic therapy was discontinued. Table S3. Antibiotic prescription at discharge and 72-h readmission according to influenza rapid diagnostic test results in patients not receiving antibiotic therapy at PED admission. Comparison of demographic characteristics, antibiotic prescription at discharge, and unplanned pediatric emergency department (PED) return visits within 72 h between influenza rapid diagnostic test (RDT)–positive and RDT-negative patients who were not receiving antibiotic therapy at presentation. Readmission rates are calculated among patients discharged without antibiotic therapy. Table S4. Antibiotic discontinuation and 72-h readmission according to influenza rapid diagnostic test results in patients receiving antibiotic therapy at PED admission. Comparison of demographic characteristics, antibiotic discontinuation at discharge, and unplanned pediatric emergency department (PED) return visits within 72 h between influenza rapid diagnostic test (RDT)–positive and RDT-negative patients who were receiving antibiotic therapy at presentation. Readmission rates are calculated among patients whose antibiotic therapy was discontinued.

## Abbreviations

The following abbreviations are used in this manuscript:

AMR	Antimicrobial Resistance
CI	Confidence Interval
OR	Odds Ratio
PED	Pediatric Emergency Departments
RDT	Rapid Diagnostic Tests
RTI	Respiratory tract infection
RT-PCR	Real-Time Polymerase Chain Reaction
TAT	Turnaround Times
