# Supplementary material for: Association Between Point-of-Care Viral Testing for Influenza and Adenovirus and Antibiotic Management in a Pediatric Emergency Department in Italy
Source: Children (Basel). 2026 Jan 21;13(1):151. doi: 10.3390/children13010151 (PMC12840226; doi:10.3390/children13010151)
Supplement: Supplementary file 1 [file children-13-00151-s001.zip › children-4068386-supplementary.pdf]

Table S1. Antibiotic prescription at discharge and 72-hour readmission according to adenovirus rapid diagnostic test results in patients not receiving antibiotic therapy at PED admission. Comparison of demographic characteristics, antibiotic prescription at discharge, and unplanned pediatric emergency department (PED) return visits within 72 hours between adenovirus rapid diagnostic test (RDT)–positive and RDT-negative patients who were not receiving antibiotic therapy at presentation. Readmission rates are calculated among patients discharged without antibiotic therapy.

	**Total Patients tested for Adenovirus n = 493**	**Positive Patients n = 45**	**Negative Patients n = 448**	**p**
**Sex, m (%)**	292 (59.2)	31 (68.8)	261	0.16
**Patients dismissed with antibiotic prescription, yes (%)**	186 (37.7)	4 (8.8)	182 (40.6)	< 0.0001
**Readmission, yes (%)***	*17/307* (5.5)	*0/41* (0)	*17/266* (6.4)	0.1

Table S2. Antibiotic discontinuation and 72-hour readmission according to adenovirus rapid diagnostic test results in patients receiving antibiotic therapy at PED admission. Comparison of demographic characteristics, antibiotic discontinuation at discharge, and unplanned pediatric emergency department (PED) return visits within 72 hours between adenovirus rapid diagnostic test (RDT)–positive and RDT-negative patients who were receiving antibiotic therapy at presentation. Readmission rates are calculated among patients whose antibiotic therapy was discontinued.

	**Total Patients tested for Adenovirus n = 109**	**Positive Patients n = 22**	**Negative Patients n = 87**	**p**
**Sex, m (%)**	60 (55)	13 (59)	47 (54)	0.67
**Suspended antibiotic therapy, yes (%)**	23 (21.1)	18	5	< 0.0001
**Readmission, yes (%)***	*2/23* (8.7)	*1/18* (5.5)	*1/5* (20)	0.37

Table S3. Antibiotic prescription at discharge and 72-hour readmission according to influenza rapid diagnostic test results in patients not receiving antibiotic therapy at PED admission. Comparison of demographic characteristics, antibiotic prescription at discharge, and unplanned pediatric emergency department (PED) return visits within 72 hours between influenza rapid diagnostic test (RDT)–positive and RDT-negative patients who were not receiving antibiotic therapy at presentation. Readmission rates are calculated among patients discharged without antibiotic therapy.

	**Total Patients tested for Influenza n = 734**	**Positive Patients n = 205**	**Negative Patients n = 529**	**p**
**Sex, m (%)**	427 (58.1)	119	308	0.96
**Patients dismissed with antibiotic prescription, yes (%)**	246 (33.5)	18	228	< 0.0001
**Readmission, yes (%)***	*29/488* (5.9)	*13/187* (6.9)	*16/301* (5.3)	0.45

Table S4. Antibiotic discontinuation and 72-hour readmission according to influenza rapid diagnostic test results in patients receiving antibiotic therapy at PED admission. Comparison of demographic characteristics, antibiotic discontinuation at discharge, and unplanned pediatric emergency department (PED) return visits within 72 hours between influenza rapid diagnostic test (RDT)–positive and RDT-negative patients who were receiving antibiotic therapy at presentation. Readmission rates are calculated among patients whose antibiotic therapy was discontinued.

	**Total Patients tested for Influenza n = 172**	**Positive Patients n = 58**	**Negative Patients n = 114**	**p**
**Sex, m (%)**	99 (57.5)	29 (50)	70 (61.4)	0.15
**Suspended antibiotic therapy, yes (%)**	55 (31.9)	41 (70.6)	14 (12.2)	< 0.0001
**Readmission, yes (%)***	*0/55* (0)	*0/41* (0)	*0/14* (0)	/
